# Risk-adapted use of thoracic radiotherapy in stage IV non-small cell lung cancer: a multicenter prognostic model study

**DOI:** 10.3389/fonc.2026.1888208

**Published:** 2026-08-26

**Authors:** Jiaxing Guo, Guangqian Ji, Jiao Zhang, Tao Zhang, Xuehua Wei, Yu Lin, Lujun Zhao

**Affiliations:** 1 Department of Radiation Oncology, Tianjin Medical University Cancer Institute and Hospital, National Clinical Research Center for Cancer, Tianjin Key Laboratory of Cancer Prevention and Therapy, Tianjin’s Clinical Research Center for Cancer, Tianjin, China; 2 Department of Radiation Oncology, The Affiliated Hospital of Inner Mongolia Medical University, Hohhot, China; 3 Department of Radiation Oncology, National Cancer Center/National Clinical Research Center for Cancer/Cancer Hospital, Chinese Academy of Medical Sciences and Peking Union Medical College, Beijing, China; 4 The Third Department of Medical Oncology, General Hospital of Ningxia Medical University, Yinchuan, Ningxia, China; 5 Department of Radiation Oncology, Sun Yat-sen University Cancer Center, Guangzhou, China

**Keywords:** immune-chemotherapy, nomogram, prognosis, stage IV non-small cell lung cancer, thoracic radiotherapy

## Abstract

**Background:**

Survival benefit of thoracic radiotherapy (TRT) in patients with stage IV non-small cell lung cancer (NSCLC) receiving first-line chemo-immunotherapy remains uncertain. This study developed and validated an overall survival (OS) prediction model and assessed whether TRT benefit varied across risk strata.

**Methods:**

Patients with stage IV NSCLC treated with first-line chemo-immunotherapy were enrolled. A nomogram was developed using baseline clinical variables to calculate individual risk scores. Patients were classified into high- and low-risk groups according to the median nomogram-derived score. OS was compared between ICI and ICI+TRT groups in the overall cohort and risk subgroups. Treatment-by-risk interaction, 1:1 propensity score matching (PSM), treatment response, TRT pattern, BED10, and metastatic burden subgroup analyses, and safety assessments were performed.

**Results:**

Among 514 patients, 284 without TRT were assigned to training and internal validation cohorts at a 7:3 ratio, and 84 non-TRT patients formed an external validation cohort. ECOG performance status, alkaline phosphatase, total protein, platelet-to-lymphocyte ratio, and systemic immune-inflammation index were selected as optimal prognostic factors. The nomogram showed good 1- and 2-year OS discrimination, calibration, and clinical utility. Using a median risk score of 103.1, TRT improved OS only in low-risk patients (HR 0.456, 95% CI 0.285–0.730; *P* = 0.0011), not high-risk patients (HR 0.914, 95% CI 0.556–1.503; *P* = 0.7239), with consistent findings after PSM. Treatment-by-risk interaction was significant (*P* = 0.0309). Grade 1–2 pneumonitis was higher with ICI+TRT than ICI alone (28.8% vs. 10.3%; *P* < 0.001), without significant differences in severe pneumonitis or immune-related adverse events.

**Conclusion:**

TRT benefit was risk-dependent, improving OS in low-risk but not high-risk patients.

## 1 Introduction

Non-small cell lung cancer (NSCLC), making up around 80%–85% of all lung cancers, poses a serious health hazard worldwide and is responsible for a large proportion of cancer-associated deaths (1). Accumulating evidence from pivotal clinical trials, including IMpower132 (2) and KEYNOTE-189 (3), has shown that immune-chemotherapy significantly improves survival outcomes in stage IV NSCLC. On this basis, immunotherapy, taken alone or with chemotherapy, has become the typical first-line therapy for patients without targetable genomic alterations.

Radiotherapy represents an important local therapeutic option for stage IV lung cancer. Specifically, in patients with synchronous oligometastatic stage IV NSCLC, thoracic radiotherapy (TRT) administered after systemic therapy has achieved disease control has been associated with improved progression-free survival and a potential overall survival (OS) benefit (4–8). However, most of these studies were conducted before the widespread adoption of immune checkpoint inhibitors and mainly focused on highly selected oligometastatic populations. In the immunotherapy era, although preclinical and clinical evidence suggests that radiotherapy may enhance antitumor immune responses and improve the efficacy of PD-1/PD-L1 inhibitors, the role of TRT in patients receiving first-line chemo-immunotherapy remains unclear. Previous studies, including PEMBRO-RT (9) and the MDACC (10) randomized trial, suggested a possible synergistic effect between radiotherapy and immunotherapy. However, these studies were limited by small sample sizes, heterogeneous treatment strategies, and insufficient identification of patients most likely to benefit from TRT. Accordingly, the central clinical question extends beyond whether TRT is associated with improved survival to whether the magnitude of this benefit varies across patients with different prognostic profiles. To our knowledge, no prior study has systematically examined the association between TRT and survival across prognostic risk strata among patients with stage IV NSCLC treated with first-line chemo-immunotherapy. Recent investigations of thoracic malignancies have shown that predictive models and machine-learning methods incorporating routinely available baseline clinical variables can support individualized prognostic evaluation and clinically relevant risk stratification (11–13). Collectively, these findings provide a methodological basis for integrating baseline host- and tumor-related characteristics to identify patient subgroups that may experience differential treatment outcomes. Therefore, there is currently no established risk-adapted strategy to guide TRT selection in unselected patients with stage IV NSCLC receiving first-line chemo-immunotherapy.

Accordingly, this retrospective study aimed to develop and validate an OS prognostic risk model for patients with stage IV NSCLC treated with first-line chemo-immunotherapy and to determine whether the association between TRT and survival differed across model-defined risk strata. Through this approach, we aimed to distinguish patients most likely to achieve a clinically meaningful survival benefit from TRT from those in whom additional thoracic local treatment may confer only limited benefit.

## 2 Materials and methods

### 2.1 Patients

This multicenter study enrolled patients with newly diagnosed stage IV NSCLC between January 2019 and December 2023 from the following institutions: Tianjin Medical University Cancer Institute and Hospital; the Affiliated Hospital of Inner Mongolia Medical University; the General Hospital of Ningxia Medical University; the National Cancer Center/National Clinical Research Center for Cancer/Cancer Hospital, Chinese Academy of Medical Sciences and Peking Union Medical College; and Sun Yat-sen University Cancer Center.

Eligibility of patients was based on the following criteria: (1) age ≥18 years; (2) stage IV disease per the eighth edition of the TNM staging system; (3) histopathologically confirmed NSCLC, including squamous cell carcinoma and adenocarcinoma; (4) an Eastern Cooperative Oncology Group (ECOG) performance status score of 0–2; and (5) receipt of first-line immune checkpoint inhibitor (ICI)-based chemo-immunotherapy, including PD-1/PD-L1 inhibitors combined with platinum-based chemotherapy.

To improve the comparability between the TRT and non-TRT groups and to reduce the influence of early death unrelated to TRT decision-making, the final analysis included only patients who survived for more than 6 months after initiation of first-line chemo-immunotherapy.

Patients were excluded if they had (1) any prior malignancy or autoimmune disease or (2) targetable driver gene alterations, including EGFR, ALK, and ROS1.

### 2.2 Data collection

Baseline clinical variables included ECOG performance status, age, smoking status, sex, histological subtype (adenocarcinoma or squamous cell carcinoma), and comorbidities, including hypertension, diabetes mellitus, and coronary artery disease; the presence of any of these conditions was considered a comorbidity. Additional variables included oligometastatic status, metastatic sites (brain, bone, liver, adrenal glands, and pleura), irAEs, and treatment response. Response was defined as the best overall response according to RECIST version 1.1, assessed during first-line treatment (ICI with or without chemotherapy), and determined before TRT initiation. Responses were categorized as complete response (CR), partial response/stable disease (PR/SD), or progressive disease (PD). OS was the primary endpoint and was calculated from the initiation of immunotherapy to death or the last follow-up.

Laboratory parameters included alkaline phosphatase (ALP), albumin, total protein (TP), white blood cell count, neutrophil count (N), lymphocyte count (L), platelet count, platelet-to-lymphocyte ratio (PLR), neutrophil-to-lymphocyte ratio (NLR), systemic inflammatory index (SII = platelet count × neutrophil count/lymphocyte count), prognostic nutritional index (PNI), and albumin-to-alkaline phosphatase ratio (AAPR).

A total of 146 patients in this study received TRT. TRT was administered after at least two cycles of first-line chemo-immunotherapy, and the specific timing of TRT initiation was determined at the discretion of the treating physician. Based on treatment timing and intent, TRT was classified as consolidative or salvage.

Consolidative TRT was defined as TRT delivered to patients who had achieved disease control (CR, PR, or SD), whereas salvage TRT referred to treatment initiated at progression. The treating radiation oncologist determined radiotherapy target volumes individually. In general, the gross tumor volume (GTV) encompassed the primary lesion, with or without metastatic mediastinal lymph nodes. The clinical target volume (CTV) included the GTV and the relevant regional drainage areas. The planning gross tumor volume was defined as the GTV plus margins to account for tumor motion and setup uncertainty, whereas the planning target volume was defined as the CTV plus margins accounting for tumor motion and setup uncertainty. Radiotherapy was delivered using conventional or hypofractionated regimens, or stereotactic body radiotherapy (SBRT). Dose regimens varied by modality, with conventional fractionation delivered at 1.8–2.2 Gy per fraction for a total dose of 40–66 Gy, hypofractionation delivered at 2.5–4.5 Gy per fraction for a total dose of 36–75 Gy, and SBRT delivered at 5–12 Gy per fraction for a total dose of 50–70 Gy. Dose constraints for organs at risk were applied in accordance with commonly accepted institutional and international radiotherapy guidelines to minimize treatment-related toxicity.

### 2.3 Data analysis and statistical considerations

Patients with missing key clinical, treatment, or follow-up data were excluded from the final analysis. Therefore, a complete case analysis approach was adopted in this study. OS served as the primary endpoint and was measured from the initiation of chemo-immunotherapy to death from any cause or the last follow-up. Patients were followed from treatment initiation until May 30, 2025. Statistical analyses were performed using R software (version 4.5).

A total of 514 patients from five centers were included in this study: 377 from Tianjin Medical University Cancer Institute and Hospital, 69 from the Affiliated Hospital of Inner Mongolia Medical University, 44 from the General Hospital of Ningxia Medical University, 10 from the Cancer Hospital, Chinese Academy of Medical Sciences, and 14 from Sun Yat-sen University Cancer Center. Among them, 368 patients did not receive TRT, whereas 146 did.

Among the 368 patients who did not receive TRT, three cohorts were established: a training, an internal validation, and an external validation cohort. Patients from Tianjin Medical University Cancer Institute and Hospital were randomly assigned in a 7:3 ratio to the training cohort (n = 200) and the internal validation cohort (n = 84). The external validation cohort (n =84) comprised patients from the other participating centers.

### 2.4 Model construction and validation

In the training cohort, optimal cutoff values for seven candidate continuous hematological variables—ALP, TP, NLR, PLR, SII, PNI, and AAPR—were identified using maximally selected rank statistics through the surv_cutpoint function in the survminer package. OS was specified as the outcome, and the minimum proportion for each group was set at 10% (minprop = 0.1). Each variable was then dichotomized into low- and high-level categories according to the corresponding data-derived cutoff. The resulting cutoff values were 108 U/L for ALP, 75.3 g/L for TP, 2.31 for NLR, 123.40 for PLR, 674.74 for SII, 48.80 for PNI, and 0.55 for AAPR. Variables with a univariable *P* value ≤0.05 were included in the initial multivariable Cox model, with no variables forced into the model based on clinical judgment. Bidirectional stepwise selection was subsequently conducted according to the Akaike information criterion. The initial model, which included seven variables, yielded an AIC of 713.8197. Exclusion of PNI decreased the AIC to 711.8591, whereas the subsequent exclusion of AAPR produced the final five-variable model, with an AIC of 711.4198. The final model included ECOG performance status, ALP, TP, PLR, and SII. These factors were then incorporated into survival prediction models for 1- and 2-year OS. Model performance was assessed by comparing the area under the receiver operating characteristic curve (AUC) and overall clinical applicability, and the optimal model was selected accordingly.

The predictive performance of the nomogram was assessed for calibration and discrimination using calibration plots, decision curve analysis (DCA), and receiver operating characteristic (ROC) curve analysis. Based on the median nomogram score, the training, internal validation, and external validation cohorts were each stratified into high- and low-risk groups. Differences in OS between the risk groups were compared using the log-rank test.

### 2.5 Prognostic risk stratification and propensity score matching (PSM)

A prognostic score for each patient was derived from the nomogram. Patients with and without TRT were each stratified into high- and low-risk groups based on their prognostic scores. Propensity score matching (PSM) was performed to reduce baseline differences between the TRT and non-TRT groups. All baseline clinical variables were included in the propensity score model. Patients were matched using a 1:1 nearest-neighbor matching algorithm with a caliper width of 0.2. Covariate balance after matching was assessed using standardized mean differences (SMDs), with an SMD < 0.1 considered indicative of adequate balance between the two groups. OS was compared between patients with and without TRT within each risk-stratified subgroup using the log-rank test.

### 2.6 Time-dependent Cox regression analysis

To mitigate potential immortal time bias arising from delayed initiation of TRT, we conducted a conditional time-dependent Cox regression analysis using 6 months after the start of first-line chemo-immunotherapy as the landmark time point. The analysis included only patients who were alive and remained under follow-up at 6 months. TRT exposure was modeled as a time-dependent covariate. Patients who initiated TRT after the landmark contributed unexposed person-time before TRT initiation and exposed person-time thereafter, whereas those who had initiated TRT by 6 months were classified as exposed from the landmark onward. Cox regression models were fitted using a counting-process formulation, with robust standard errors clustered according to patient ID. An interaction term between TRT and risk group was included to evaluate whether the association between TRT and OS varied among risk groups.

## 3 Results

### 3.1 Baseline characteristics

The baseline clinical characteristics of all enrolled patients are presented in **Table 1**. The median follow-up duration was 30.8 months. During follow-up, 153 patients (41.6%) reached the primary endpoint. The 1-, 2-, and 3-year OS rates in the entire cohort were 91.9%, 64.9%, and 46.7%, respectively. The median OS was 37.4, 31.3, and 32.6 months in the training, internal validation, and external validation cohorts, respectively, with no statistically significant differences among the three groups (*P* = 0.37).

**Table 1:** Baseline patient characteristics.

Variables	Cutoff	Non-TRT (N = 368)			TRT (N = 146)
		Training cohort (N = 200)	Internal validation cohort(N = 84)	External cohort (N = 84)	
Age					
< 70		163 (81.5%)	70 (83.3%)	53 (63.1%)	122 (83.6%)
≥ 70		37 (18.5%)	14 (16.7%)	31 (36.9%)	24 (16.4%)
Sex					
Female		39 (19.5%)	18 (21.4%)	15 (17.9%)	29 (19.9%)
Male		161 (80.5%)	66 (78.6%)	69 (82.1%)	117 (80.1%)
N stage					
N0-1		25 (12.5%)	9 (10.7%)	16 (19%)	20 (13.7%)
N2-3		175 (87.5%)	75 (89.3%)	68 (81%)	126 (86.3%)
T stage					
T1-2		96 (48%)	36 (42.9%)	30 (35.7%)	67 (45.9%)
T3-4		104 (52%)	48 (57.1%)	54 (64.3%)	79 (54.1%)
Oligo					
No		170 (85%)	72 (85.7%)	67 (79.8%)	92 (63%)
Yes		30 (15%)	12 (14.3%)	17 (20.2%)	54 (37%)
Liver metastasis					
No		182 (91%)	79 (94%)	74 (88.1%)	139 (95.2%)
Yes		18 (9%)	5 (6%)	10 (11.9%)	7 (4.8%)
Pleural effusion					
No		159 (79.5%)	62 (73.8%)	73 (86.9%)	134 (91.8%)
Yes		41 (20.5%)	22 (26.2%)	11 (13.1%)	12 (8.2%)
Brain metastases					
No		174 (87%)	76 (90.5%)	74 (88.1%)	120 (82.2%)
Yes		26 (13%)	8 (9.5%)	10 (11.9%)	26 (17.8%)
Bone metastases					
No		123 (61.5%)	56 (66.7%)	45 (53.6%)	99 (67.8%)
Yes		77 (38.5%)	28 (33.3%)	39 (46.4%)	47 (32.2%)
Adrenal metastasis					
No		176 (88%)	76 (90.5%)	72 (85.7%)	132 (90.4%)
Yes		24 (12%)	8 (9.5%)	12 (14.3%)	14 (9.6%)
Pleura involvement					
No		167 (83.5%)	64 (76.2%)	61 (72.6%)	120 (82.2%)
Yes		33 (16.5%)	20 (23.8%)	23 (27.4%)	26 (17.8%)
Basic Disease					
No		121 (60.5%)	53 (63.1%)	49 (58.3%)	86 (58.9%)
Yes		79 (39.5%)	31 (36.9%)	35 (41.7%)	60 (41.1%)
Smoking					
No		62 (31%)	26 (31%)	43 (51.2%)	61 (41.8%)
Yes		138 (69%)	58 (69%)	41 (48.8%)	85 (58.2%)
ECOG					
0-1		192 (96%)	77 (91.7%)	75 (89.3%)	138 (94.5%)
2		8 (4%)	7 (8.3%)	9 (10.7%)	8 (5.5%)
Histology					
Adenocarcinoma		127 (63.5%)	58 (69%)	35 (41.7%)	66 (45.2%)
Squamous		73 (36.5%)	26 (31%)	49 (58.3%)	80 (54.8%)
PDL-1					
Negative		20 (10%)	7 (8.3%)	5 (6%)	22 (15.1%)
Positive		68 (34%)	22 (26.2%)	25 (29.8%)	35 (24%)
Unclear		112 (56%)	55 (65.5%)	54 (64.3%)	89 (61%)
ALP					
Low	≤108 U/L	152 (76%)	54 (64.3%)	59 (70.2%)	95 (65.1%)
High	>108 U/L	48 (24%)	30 (35.7%)	25 (29.8%)	51 (34.9%)
TP					
Low	≤ 75.3 g/L	163 (81.5%)	74 (88.1%)	73 (86.9%)	107 (73.3%)
High	> 75.3 g/L	37 (18.5%)	10 (11.9%)	11 (13.1%)	39 (26.7%)
NLR					
Low	≤ 2.31	56 (28%)	22 (26.2%)	21 (25%)	39 (26.7%)
High	> 2.31	144 (72%)	62 (73.8%)	63 (75%)	107 (73.3%)
PLR					
Low	≦ 123.40	37 (18.5%)	14 (16.7%)	18 (21.4%)	31 (21.2%)
High	> 123.40	163 (81.5%)	70 (83.3%)	66 (78.6%)	115 (78.8%)
SII					
Low	≤ 674.74	70 (35%)	26 (31%)	30 (35.7%)	47 (32.2%)
High	> 674.74	130 (65%)	58 (69%)	54 (64.3%)	99 (67.8%)
PNI					
Low	≤ 48.80	91 (45.5%)	45 (53.6%)	56 (66.7%)	59 (40.4%)
High	> 48.80	109 (54.5%)	39 (46.4%)	28 (33.3%)	87 (59.6%)
AAPR					
Low	≤ 0.55	157 (78.5%)	62 (73.8%)	58 (69%)	100 (68.5%)
High	> 0.55	43 (21.5%)	22 (26.2%)	26 (31%)	46 (31.5%)

### 3.2 Variable selection and model construction

**Figure 1:**
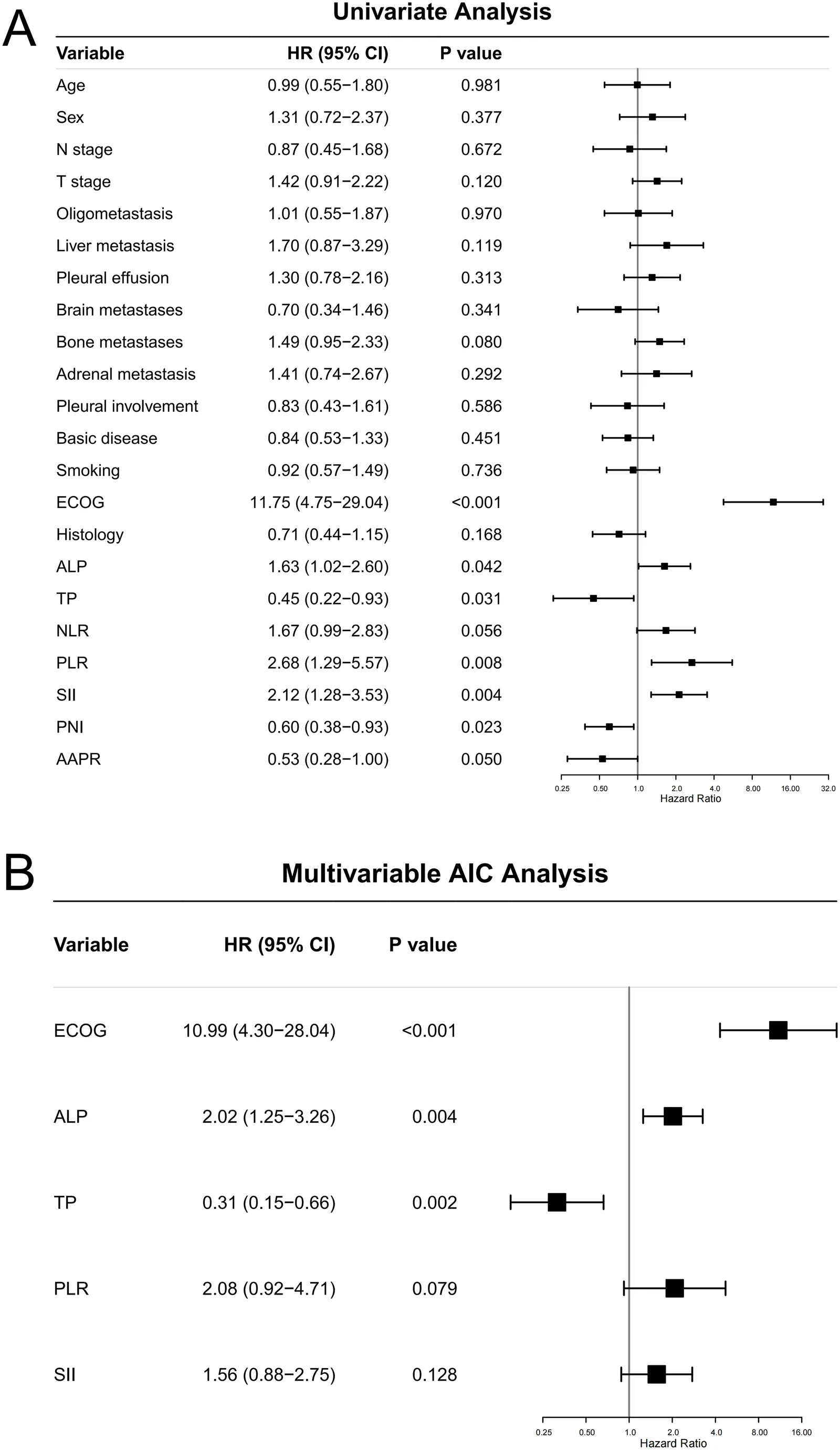
Forest plots of Cox regression analyses for overall survival. **(A)** Univariable Cox analysis of candidate prognostic factors, presented as hazard ratios (HRs) with 95% confidence intervals (CIs) and corresponding *P* values. **(B)** Multivariable Cox model selected based on the Akaike information criterion (AIC), with HRs, 95% CIs, and P values for retained variables.

**Figure 2:**
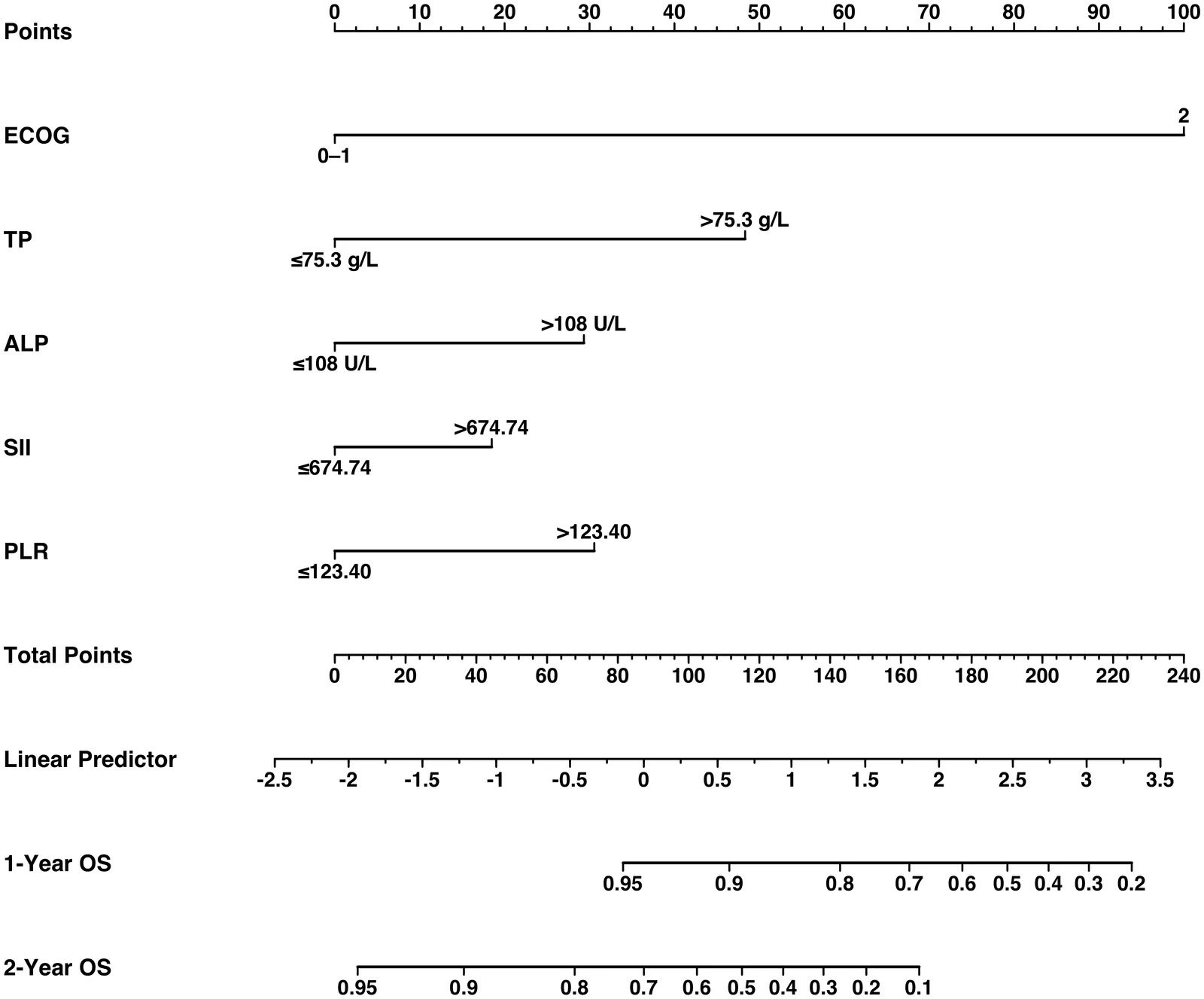
Nomogram for predicting overall survival in patients with stage IV NSCLC after first-line chemo-immunotherapy. The nomogram was developed to predict overall survival (OS) in patients with stage IV non-small cell lung cancer (NSCLC) after receiving first-line chemo-immunotherapy. The final model incorporated Eastern Cooperative Oncology Group (ECOG) performance status, total protein (TP), alkaline phosphatase (ALP), systemic immune-inflammation index (SII), and platelet-to-lymphocyte ratio (PLR). ECOG performance status 2 was assigned 100.0 points; ALP > 108 U/L was assigned 29.3 points; TP ≤75.3 g/L was assigned 48.3 points; PLR > 123.40 was assigned 30.6 points; SII > 674.74 was assigned 18.5 points; and other conditions were assigned 0.0 points. A vertical line was drawn upward from the corresponding value of each variable to determine the assigned points, and the points from all variables were summed to obtain the total score. For each patient with stage IV NSCLC, the total points were projected downward to the outcome scale to estimate the predicted probabilities of 1-year and 2-year OS.

To identify prognostic factors for nomogram construction, univariable analyses were first performed in the training cohort, and variables with *P ≤* 0.05 were considered for multivariable modeling as described in the Methods (**Figures 1A, B**). ECOG, ALP, TP, PLR, and SII were retained in the final prognostic model. A prognostic nomogram was subsequently developed based on these variables (**Figure 2**).

### 3.3 Model evaluation and validation

**Figure 3:**
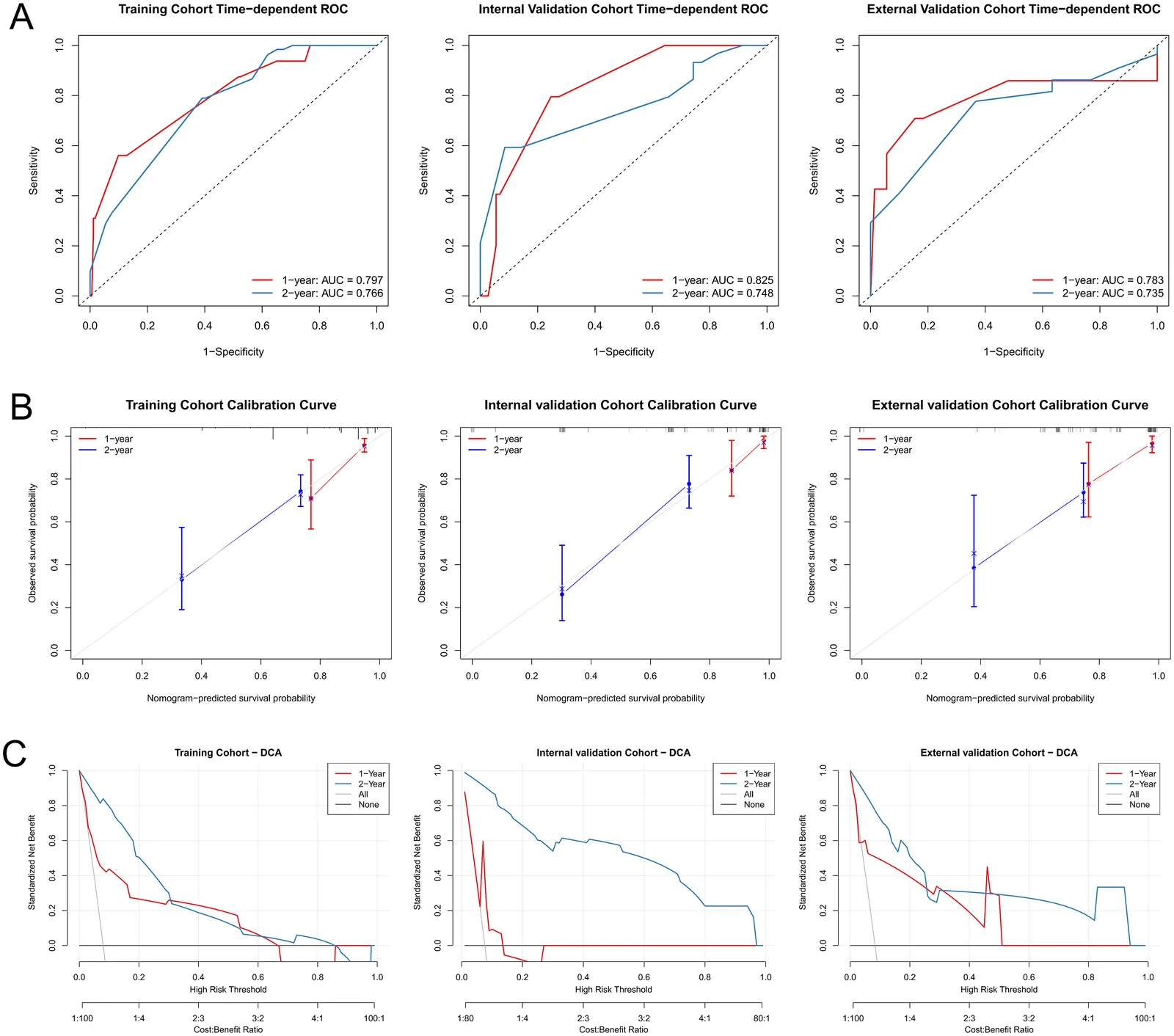
**(A)** Receiver operating characteristics (ROC) curve in training, internal validation, and external validation cohorts; AUC, area under the ROC. **(B)** Calibration curves of the nomogram in the same cohorts. **(C)** Decision curve analysis for the nomogram in the same cohorts.

The robustness of the nomogram was assessed using ROC analysis, calibration plots, and DCA. In the training cohort, the time-dependent AUCs for predicting 1- and 2-year OS were 0.797 (95% CI, 0.683–0.910) and 0.766 (95% CI, 0.694–0.838), respectively. In the internal validation cohort, the corresponding AUCs were 0.825 (95% CI, 0.679–0.972) and 0.748 (95% CI, 0.628–0.867), whereas those in the external validation cohort were 0.783 (95% CI, 0.533–1.000) and 0.735 (95% CI, 0.594–0.875), respectively, indicating generally consistent discriminatory performance across the three cohorts (**Figure 3A**; **Table 2**). At the 1-year prediction horizon, the calibration intercepts were 0.117, −0.949, and −0.596 for the training, internal validation, and external validation cohorts, respectively, whereas the corresponding calibration slopes were 1.055, 0.765, and 0.893. At 2 years, the calibration intercepts were 0.369, 0.135, and −0.186, respectively, and the corresponding slopes were 1.456, 1.165, and 0.865. The Brier scores were 0.063, 0.075, and 0.062 at 1 year and 0.173, 0.183, and 0.175 at 2 years in the training, internal validation, and external validation cohorts, respectively. Overall, the model demonstrated broadly consistent discrimination and prediction error across all three cohorts. However, departures from ideal calibration were evident at certain prediction horizons (**Table 2**). In addition, calibration curves for the training, internal validation, and external validation cohorts showed that predicted and observed survival probabilities agreed well, suggesting satisfactory calibration performance (**Figure 3B**). Moreover, DCA demonstrated a consistently positive net benefit across a wide range of threshold probabilities, further supporting the favorable clinical utility of this prognostic model (**Figure 3C**).

**Table 2:** Performance of the prognostic model in the training, internal validation, and external validation cohorts.

Cohort	Prediction horizon	AUC (95% CI)	Calibration intercept	Calibration slope	Brier score
Training cohort	1 year	0.797 (0.683–0.910)	0.117	1.055	0.063
Training cohort	2 years	0.766 (0.694–0.838)	0.369	1.456	0.173
Internal validation cohort	1 year	0.825 (0.679–0.972)	-0.949	0.765	0.075
Internal validation cohort	2 years	0.748 (0.628–0.867)	0.135	1.165	0.183
External validation cohort	1 year	0.783 (0.533–1.000)	-0.596	0.893	0.062
External validation cohort	2 years	0.735 (0.594–0.875)	-0.186	0.865	0.175

AUCs were estimated using time-dependent receiver operating characteristic analysis. Calibration intercepts and slopes were evaluated at 1 and 2 years; ideal calibration corresponds to an intercept of 0 and a slope of 1. Brier scores were calculated using inverse probability of censoring weighting, with lower values indicating lower prediction error. AUC, area under the receiver operating characteristic curve; CI, confidence interval; OS, overall survival.

### 3.4 Risk stratification

**Figure 4:**
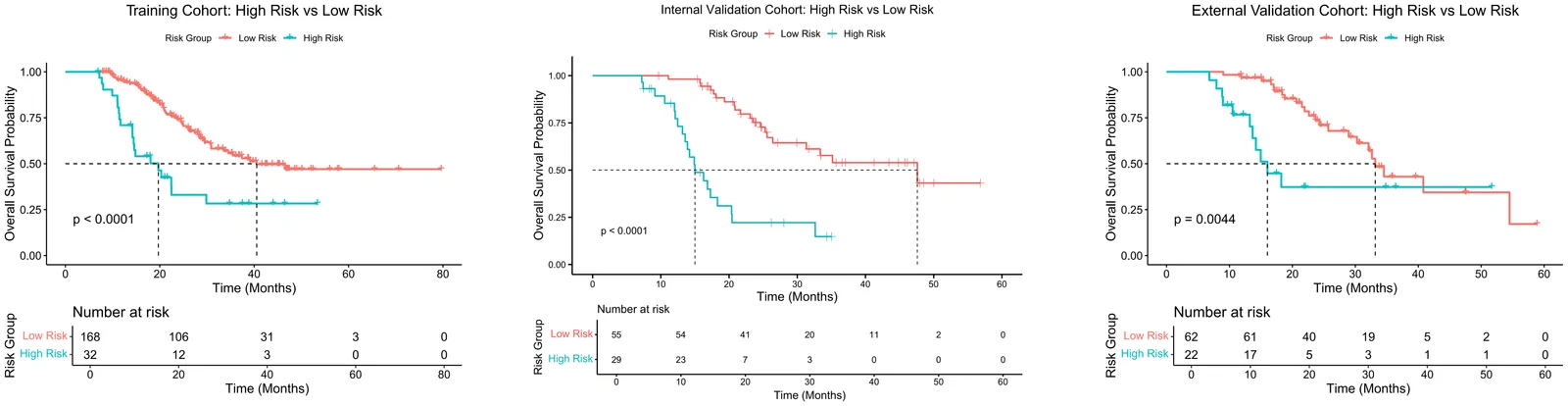
Kaplan-Meier curves for overall survival (OS) according to risk stratification in the training, internal validation, and external validation cohorts.

Using a median risk score of 103.1 derived from the training cohort, patients in the training, internal validation, and external validation cohorts were stratified into high- and low-risk groups. In the training cohort, 168 of 200 patients (84.0%) were assigned to the low-risk group, whereas 32 (16.0%) were categorized as high risk. In the internal validation cohort, 55 of 84 patients (65.5%) were classified as low risk and 29 (34.5%) as high risk; the corresponding numbers in the external validation cohort were 62 of 84 (73.8%) and 22 of 84 (26.2%), respectively. Relative to patients in the low-risk group, those in the high-risk group had a significantly greater risk of death in the training cohort (HR, 2.783; 95% CI, 1.672–4.635; *P* < 0.001), the internal validation cohort (HR, 5.031; 95% CI, 2.625–9.642; *P* < 0.001), and the external validation cohort (HR, 2.788; 95% CI, 1.336–5.820; *P* = 0.0063). Kaplan–Meier survival curves demonstrated statistically significant differences in OS between the high- and low-risk groups across all three cohorts (log-rank *P* < 0.0001, *P* < 0.0001, and *P* = 0.0044, respectively), indicating consistent prognostic separation using the fixed training-derived cutoff. (**Figure 4**; **Supplementary Table 1**).

As an exploratory *post hoc* sensitivity analysis, we additionally assessed tertile-based risk stratification using cutoff values derived solely from the training cohort and subsequently applied without modification to the internal and external validation cohorts. The thresholds corresponding to the 33.3rd and 66.7th percentiles were 83.17 and 103.1, respectively. The three tertile-defined risk strata showed significant overall separation in OS in the training, internal validation, and external validation cohorts, with log-rank *P* values of < 0.0001, < 0.0001, and 0.0065, respectively. Mortality risk also increased significantly and progressively across the ordered risk categories in all three cohorts, with *P* values for trend of < 0.001, < 0.001, and 0.0027, respectively. However, discrimination between the intermediate- and low-tertile groups was less consistent in the two validation cohorts (**Supplementary Table 2**; **Supplementary Figure 1**).

### 3.5 Prognostic value of TRT for low- and high- risk patients

**Figure 5:**
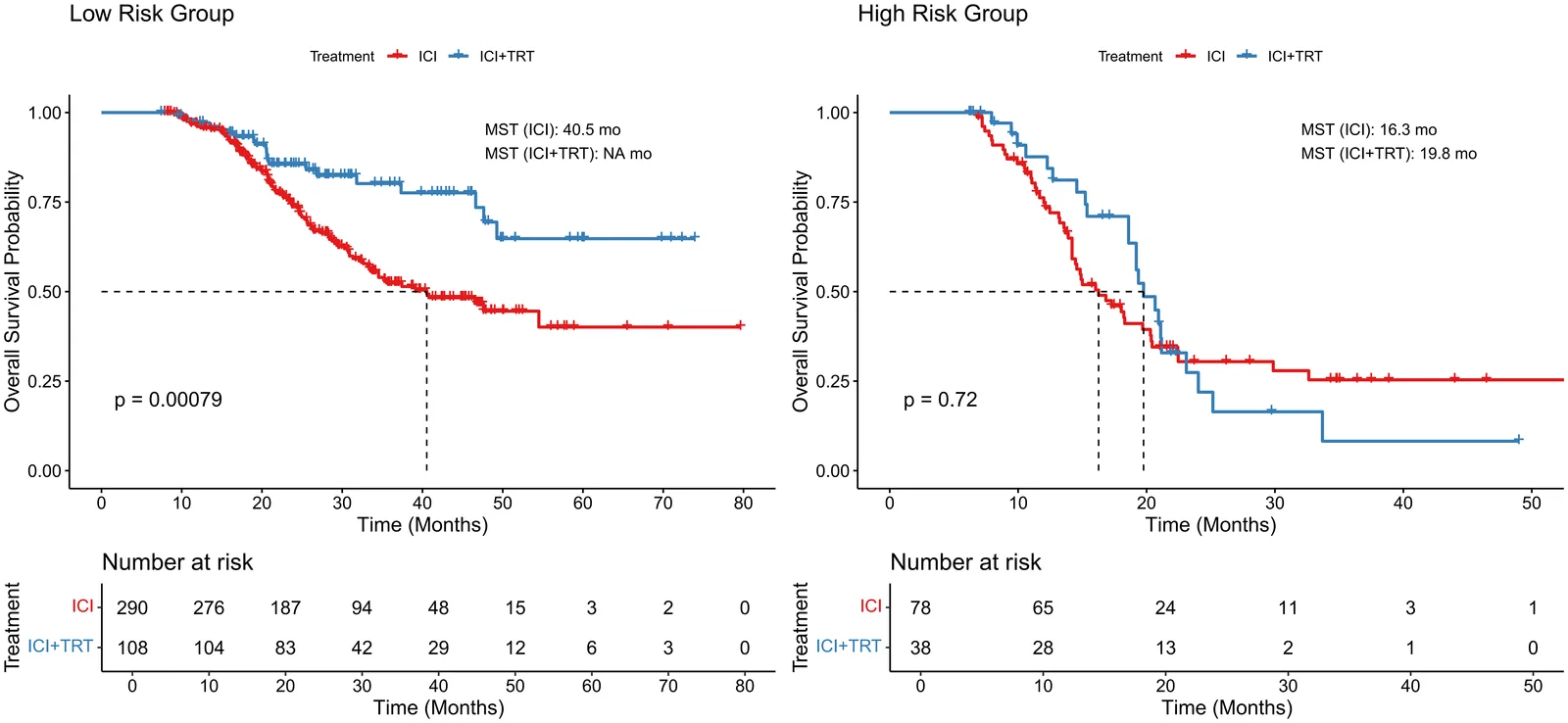
Kaplan-Meier curves for overall survival (OS) in low-risk and high-risk groups, comparing TRT versus no TRT before PSM.

All patients were grouped into high- and low-risk categories according to the median nomogram-derived risk score, regardless of TRT exposure. In the low-risk group, TRT was associated with significantly improved OS (*P* = 0.00079). In contrast, TRT did not confer a survival benefit in the high-risk group (*P* = 0.72) (**Figure 5**). To assess whether the effect of TRT varied across risk strata defined by the OS prediction model, an interaction analysis was conducted in the overall cohort. Overall, ICI+TRT was associated with significantly better OS than ICI alone (HR 0.657, 95% CI 0.469–0.919, *P* = 0.0141). This association remained significant in the low-risk group (HR 0.456, 95% CI 0.285–0.730, *P* = 0.0011), but not in the high-risk group (HR 0.914, 95% CI 0.556–1.503, *P* = 0.7239). The treatment-by-risk interaction was statistically significant (*P* for interaction = 0.0309), suggesting heterogeneity in the association between TRT and OS across risk groups (**Table 3**).

**Table 3:** Interaction analysis of the association between TRT and OS according to prognostic risk group.

Risk group	ICI (N)	ICI+TRT (N)	HR (ICI+TRT vs ICI)	95% CI	*P* value	*P* for Interaction
Overall	368	146	0.657	0.469–0.919	0.0141	0.0309
Low risk Group	290	108	0.456	0.285–0.730	0.0011	
High risk Group	78	38	0.914	0.556–1.503	0.7239	

HR < 1 indicates a lower risk of death for ICI+TRT versus ICI alone. The interaction *P* value assesses whether the treatment effect of TRT differs across OS risk groups.

To further mitigate potential immortal time bias associated with delayed TRT initiation, we conducted a conditional time-dependent Cox regression analysis using 6 months after the initiation of first-line chemo-immunotherapy as the landmark time point. In the overall cohort, TRT was associated with a reduced risk of death; however, this association did not reach statistical significance (HR, 0.752; 95% CI, 0.543–1.041; *P* = 0.086). Among patients in the low-risk group, TRT remained significantly associated with improved OS (HR, 0.537; 95% CI, 0.336–0.860; *P* = 0.010), whereas no significant association was detected in the high-risk group (HR, 1.095; 95% CI, 0.709–1.692; *P* = 0.682). The time-dependent interaction between treatment and risk group also remained statistically significant (*P* for interaction = 0.047), indicating potential heterogeneity in the association between TRT and OS across prognostic risk groups (**Table 4**).

**Table 4:** Conditional time-dependent Cox analysis of thoracic radiotherapy from the 6-month landmark.

Risk group	Never received TRT (N)	TRT initiated by 6 months (N)	TRT initiated after 6 months (N)	HR (95% CI)	*P* value	*P* for interaction
Overall	368	86	60	0.752 (0.543–1.041)	0.086	0.047
Low risk Group	290	61	47	0.537 (0.336–0.860)	0.010	
High risk Group	78	25	13	1.095 (0.709–1.692)	0.682	

1. TRT was modeled as a time-dependent covariate. Patients who had initiated TRT by 6 months were considered exposed from the landmark onward.

2. Patients initiating TRT after 6 months contributed unexposed person-time before TRT initiation and exposed person-time thereafter.

3. Patients who never received TRT remained unexposed throughout follow-up.

4. HR < 1 favors TRT.

5. *P* for interaction was derived from the combined model containing the time-dependent TRT-by-risk-group interaction term.

**Figure 6:**
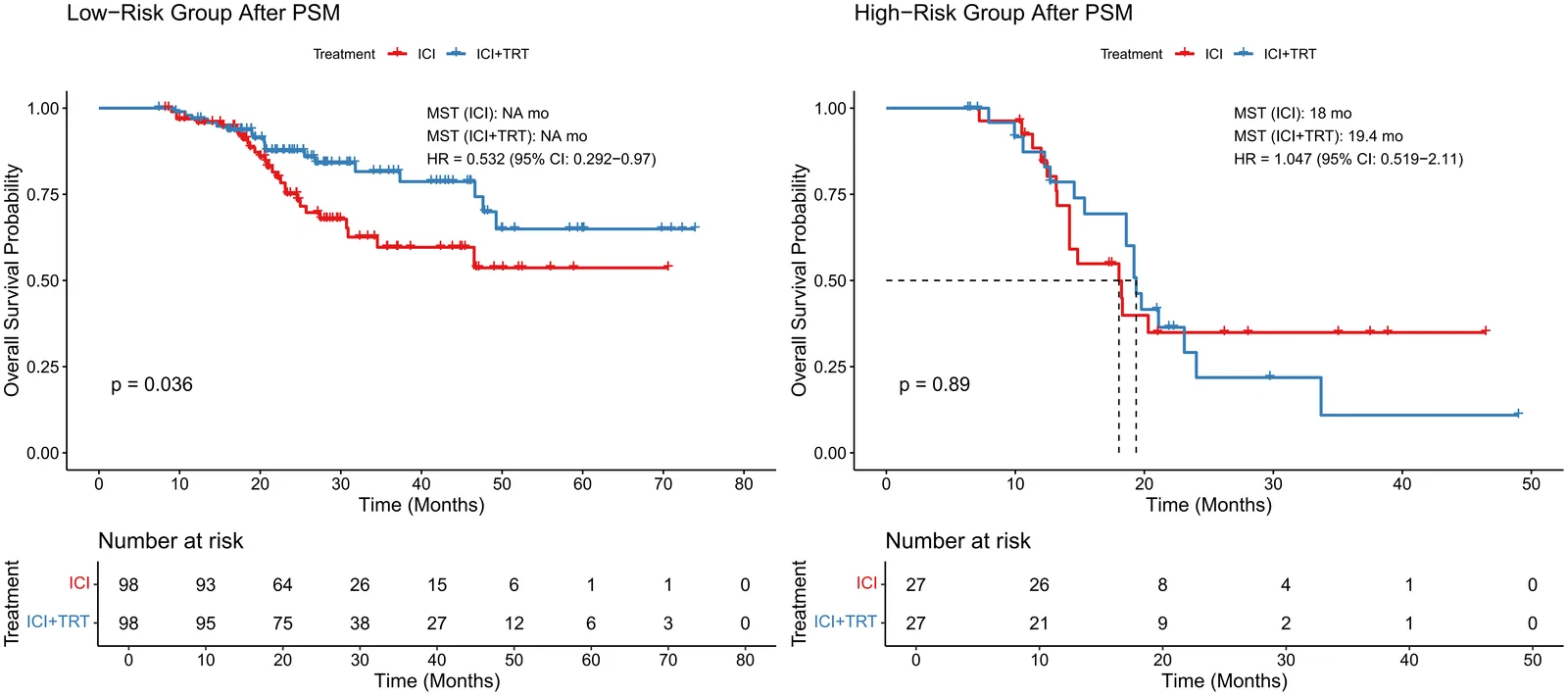
Kaplan-Meier curves for overall survival (OS) in low-risk and high-risk groups, comparing TRT versus no TRT after PSM.

To minimize potential confounding, 1:1 propensity score matching (PSM) was performed within each risk group using all baseline clinical variables as matching covariates, including age, sex, N stage, T stage, oligometastatic status, and all other baseline clinicopathological variables. Nearest-neighbor matching was applied with a caliper width of 0.1. Covariate balance before and after matching was assessed using standardized mean differences (SMDs), with an SMD < 0.1 indicating adequate balance. After PSM, baseline characteristics between the TRT and non-TRT groups were well balanced, with all SMDs below 0.1. In the low-risk group, TRT remained significantly associated with improved OS after matching (HR = 0.532, 95% CI: 0.292–0.970; log-rank *P* = 0.036). At 1 year, the OS rate was 96.9% in both the ICI+TRT and ICI groups, indicating no clinically meaningful absolute difference in OS. By 2 years, the corresponding OS rates were 87.6% and 75.1%, yielding an absolute difference of 12.5 percentage points and an exploratory NNT-like estimate of 9. In contrast, no significant survival benefit was observed in the high-risk group after PSM (HR = 1.047, 95% CI: 0.519–2.110; log-rank *P* = 0.89). These findings further support the risk-dependent survival benefit of TRT (**Figure 6**). The baseline characteristics of patients in the high- and low-risk groups before and after PSM are presented in **Supplementary Tables 3**, **4** and **Supplementary Figure 2**. Detailed landmark-specific OS estimates are presented in **Supplementary Table 5**.

Based on the findings of this study, we developed a comprehensive online tool designed to provide individualized clinical decision support for patients with stage IV NSCLC receiving first-line chemo-immunotherapy. The platform not only estimates 1- and 2-year OS probabilities but also incorporates an automated risk-stratification module. Using the derived linear predictor, a nomogram score was calculated for each patient, and a predefined cutoff value of 103.1 was applied to classify patients into high- and low-risk groups. This dual-function system enables clinicians to obtain survival predictions and risk classification simultaneously, thereby helping determine whether TRT may be appropriate and facilitating real-time clinical decision-making. The online tool is available at: https://jiaxing-predictive-model.shinyapps.io/jiaxing-predictive-model/.

### 3.6 Subgroup analyses

**Figure 7:**
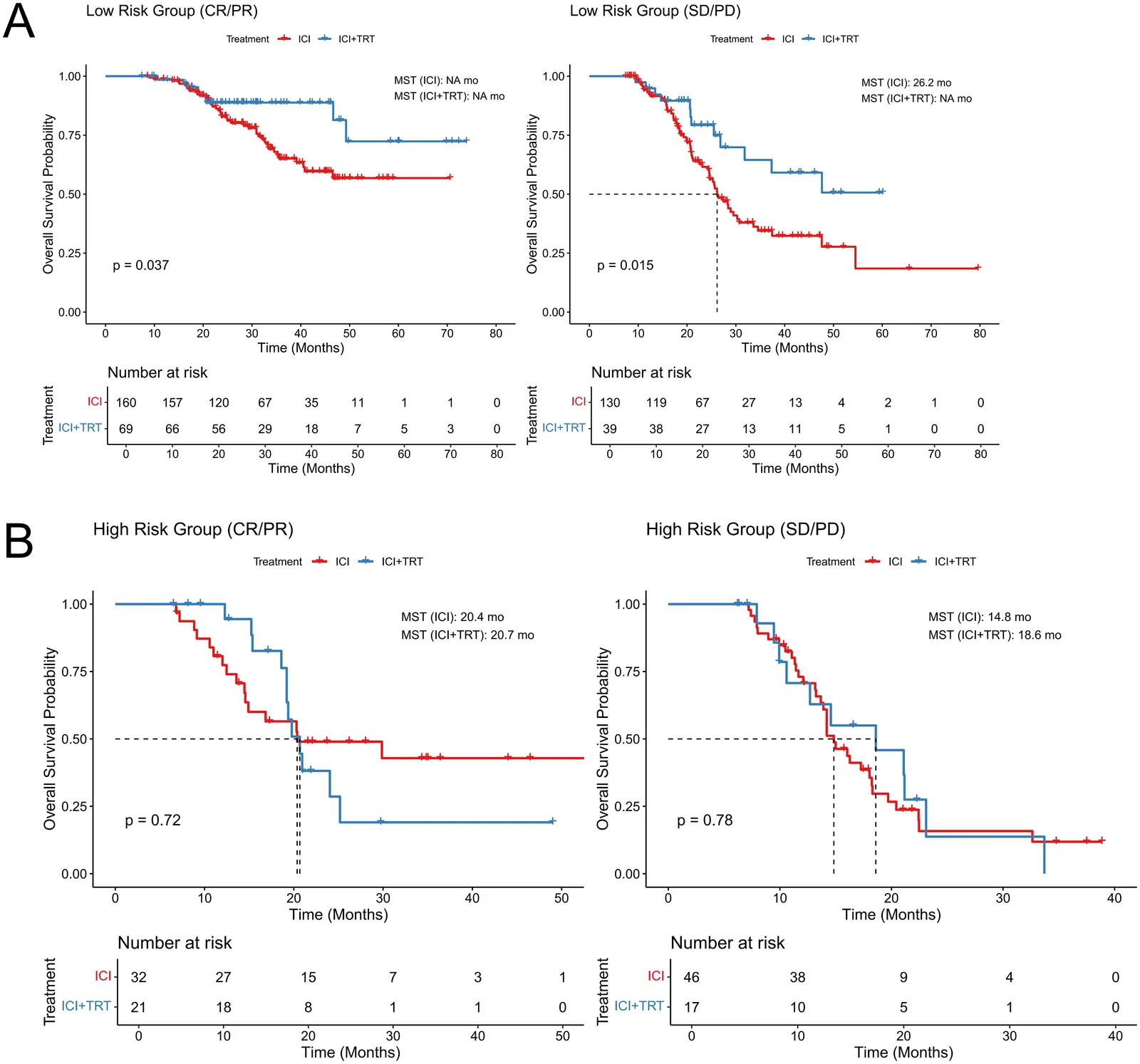
**(A)** Kaplan-Meier curves for overall survival (OS) according to TRT in the low-risk group, stratified by treatment response. **(B)** Kaplan-Meier curves for OS according to TRT in the high-risk group, stratified by treatment response.

To further explore the potential benefit of TRT, subgroup analyses were conducted within the overall cohort, stratified by both nomogram-defined risk group and treatment response. In the low-risk group, patients receiving ICI+TRT showed significantly better OS than those receiving ICI alone across response subgroups (CR/PR: *P* = 0.037; SD/PD: *P* = 0.015). In contrast, no significant differences in OS were observed between treatment groups within the high-risk group, regardless of response status (CR/PR: *P* = 0.72; SD/PD: *P* = 0.78) (**Figure 7**). Overall, these findings indicate that the survival benefit of TRT was more evident in low-risk patients and persisted regardless of treatment response status.

**Figure 8:**
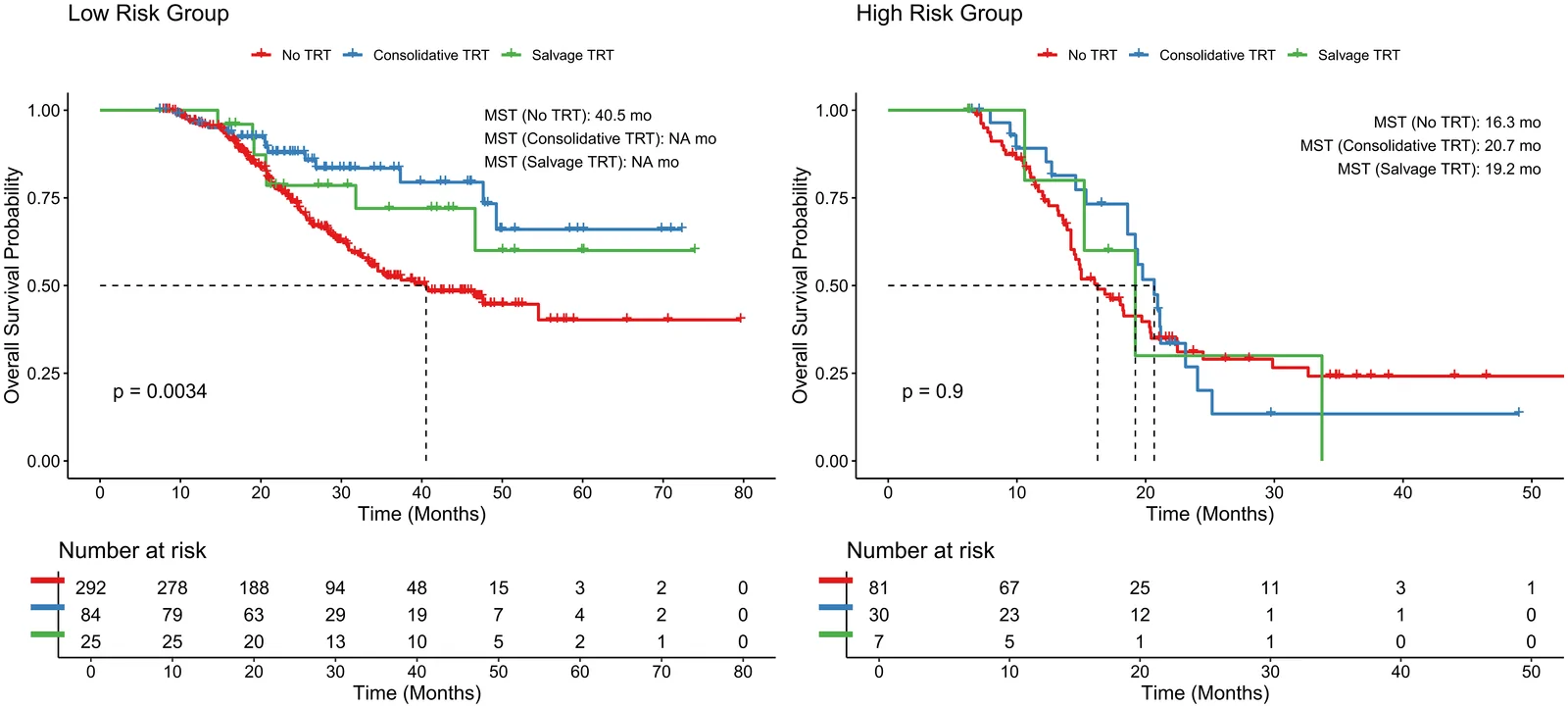
Kaplan-Meier curves for overall survival (OS) by TRT pattern in low- and high-risk groups.

To further evaluate the efficacy of different TRT patterns across risk strata, patients were categorized into no TRT, consolidative TRT, and salvage TRT groups, and survival analyses were conducted individually in the low-risk and high-risk groups. In the low-risk group, OS differed significantly among the three TRT patterns (*P* = 0.0034). Compared with patients who did not receive TRT, those who underwent consolidative or salvage TRT showed a more favorable survival. The MST was 40.5 months in the no-TRT group, whereas the MST was not reached in either the consolidative TRT group or the salvage TRT group. In the high-risk group, OS did not differ significantly among the three treatment patterns (*P* = 0.90) (**Figure 8**). Exploratory analyses of treatment timing revealed no statistically significant differences in OS between early and late consolidative TRT or between early and late salvage TRT among patients in the low-risk group (log-rank *P* = 0.2418 and *P* = 0.1362, respectively; **Supplementary Figure 3**). Because salvage TRT was administered at the time of disease progression rather than allocated at baseline, these results should be interpreted as exploratory and may be subject to confounding by indication and immortal time bias.

We also explored whether the association between TRT and survival varied according to the biologically effective dose. BED10 data were available for all 146 patients who received TRT. The median BED10 was 72.00 Gy (IQR, 61.80–75.00) in the low-risk group and 70.20 Gy (IQR, 60.00–72.00) in the high-risk group, with no statistically significant difference between the two groups (*P* = 0.131).

**Figure 9:**
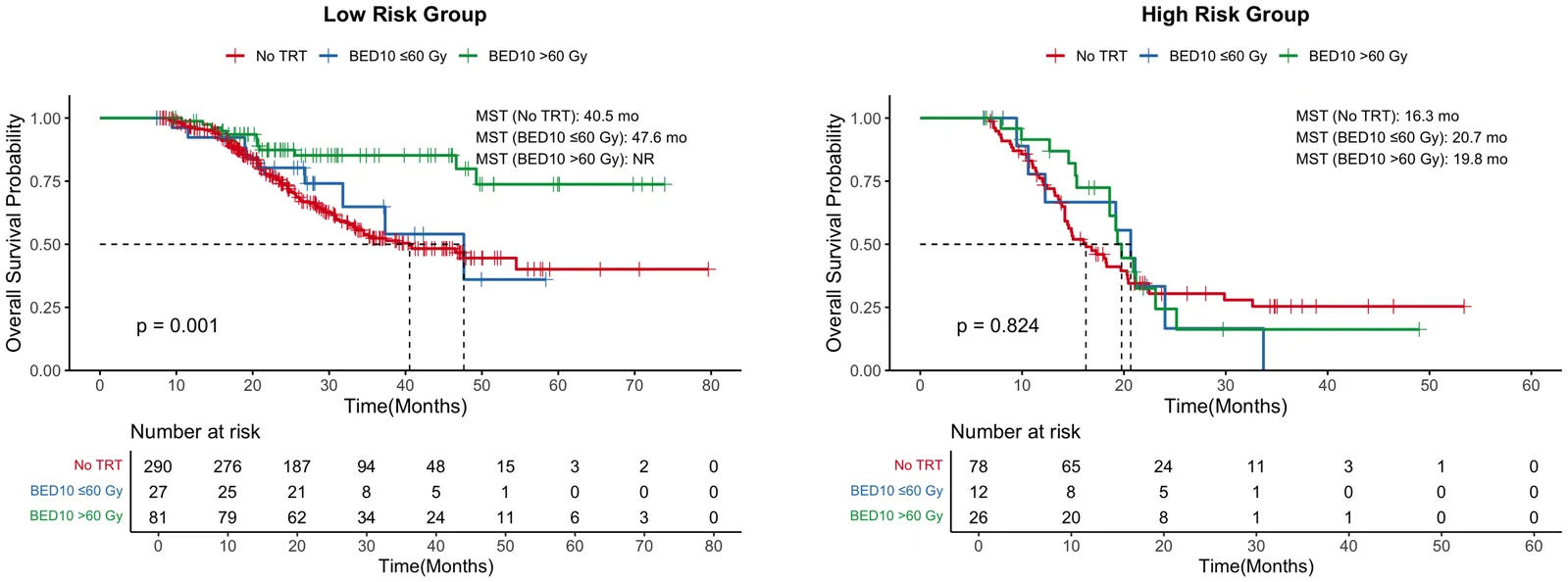
Kaplan-Meier curves for overall survival (OS) according to BED10 category in low- and high-risk groups.

A BED10 greater than 60 Gy was delivered to 75.0% of patients in the low-risk group and 68.4% of those in the high-risk group, with no statistically significant difference between the groups (*P* = 0.430; **Supplementary Table 6**). Patients who received TRT were further categorized according to BED10 as ≤60 Gy or >60 Gy, and survival outcomes in these two categories were compared with those of patients who did not receive TRT within each risk group. Among patients in the low-risk group, OS differed significantly across the no-TRT, BED10 ≤60 Gy, and BED10 >60 Gy categories (*P* = 0.001). The median survival time was 40.5 months in the no-TRT group and 47.6 months in the BED10 ≤60 Gy group, whereas the median was not reached in the BED10 >60 Gy group. In contrast, no significant difference in OS was detected across the three BED10 categories in the high-risk group (*P* = 0.824). The corresponding median survival times were 16.3, 20.7, and 19.8 months for the no-TRT, BED10 ≤60 Gy, and BED10 >60 Gy groups, respectively (**Figure 9**).

We further investigated whether metastatic burden modified the association between TRT and survival within the low- and high-risk groups. In the low-risk group, TRT was associated with a reduced risk of death in both metastatic-burden subgroups. Among patients with oligometastatic disease, the HR for ICI+TRT versus ICI alone was 0.522 (95% CI, 0.239–1.140; *P* = 0.103). Among those with polymetastatic disease, ICI+TRT was significantly associated with improved OS (HR, 0.399; 95% CI, 0.213–0.747; *P* = 0.004). However, the interaction between treatment and metastatic burden was not statistically significant in the low-risk group (*P* for interaction = 0.605), indicating no clear evidence that metastatic burden modified the association between TRT and OS.

In the high-risk group, TRT was not significantly associated with OS in either metastatic-burden subgroup. The HR for ICI+TRT versus ICI alone was 1.322 (95% CI, 0.414–4.224; *P* = 0.638) among patients with oligometastatic disease and 0.858 (95% CI, 0.483–1.525; *P* = 0.602) among those with polymetastatic disease. Similarly, no statistically significant interaction between treatment and metastatic burden was detected in the high-risk group (*P* for interaction = 0.616).

**Figure 10:**
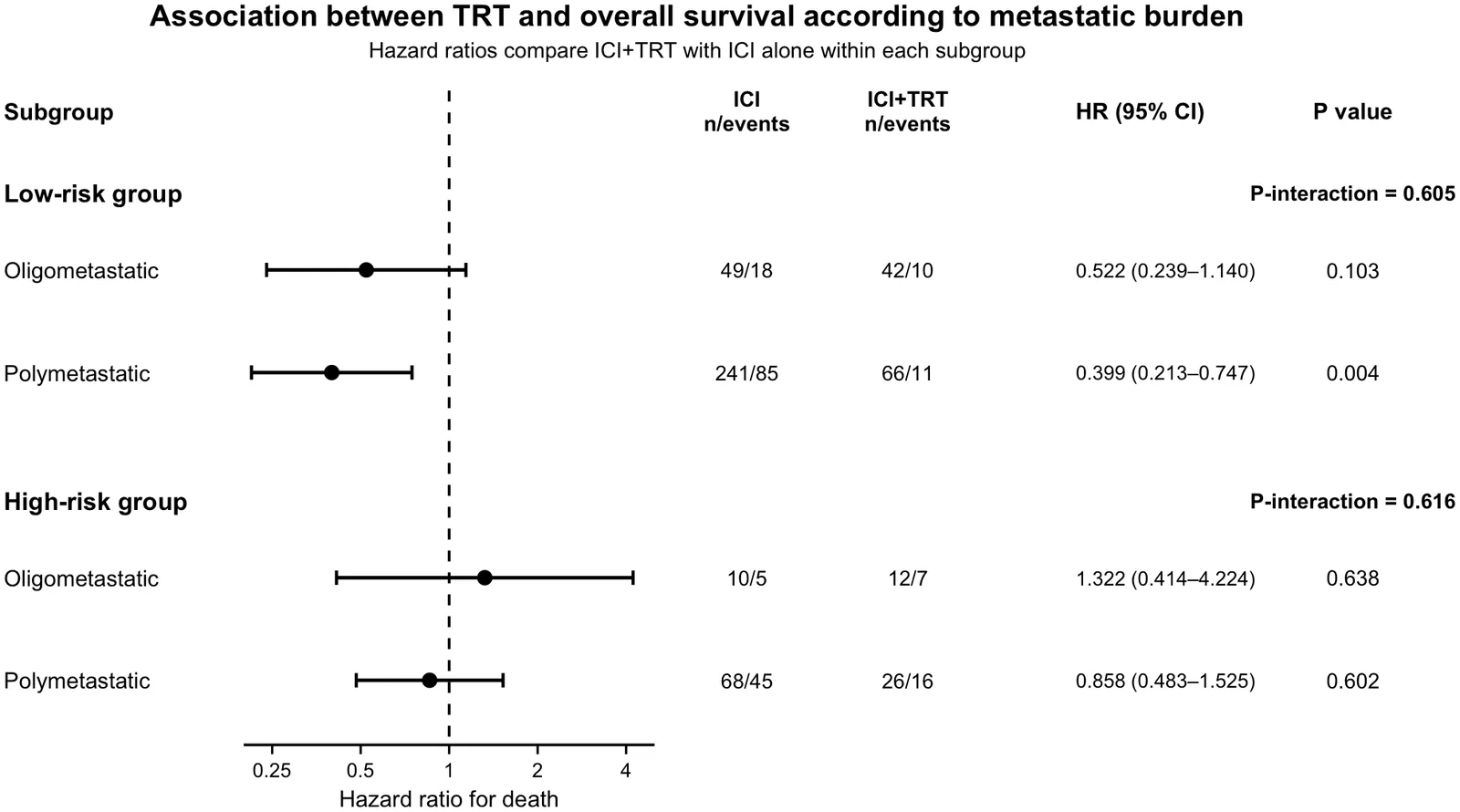
Association between thoracic radiotherapy and overall survival according to prognostic risk group and metastatic burden.

No statistically significant evidence indicated that metastatic burden modified the association between TRT and OS within either prognostic risk group (**Figure 10**). Nevertheless, the relatively small sample sizes and wide confidence intervals in the oligometastatic subgroups limit the strength of these findings. Accordingly, the results should be regarded as exploratory and should not be interpreted as definitive evidence that the association between TRT and OS is consistent irrespective of metastatic burden.

### 3.7 Treatment-related adverse events

The incidence of grade 1–2 pneumonitis was significantly higher in the ICI+TRT group than in the ICI group (28.8% vs. 10.3%, *P* < 0.001), whereas no significant difference was observed in the incidence of grade 3–4 pneumonitis (4.1% vs. 1.6%, *P* = 0.1755). The overall incidences of grade 1–2 irAEs (43.2% vs. 39.1%, *P* = 0.4603) and grade 3 irAEs (6.8% vs. 4.6%, *P* = 0.4222) were comparable between the two groups. Similarly, no significant differences were observed in endocrine toxicity, dermatitis, or hepatitis. Grade 3 endocrine toxicity was not reported in either group (**Table 5**).

**Table 5:** TRAEs between the two treatment groups.

TRAE	ICI(N = 368)	ICI+TRT(N = 146)	*P* value
G1–2 irAEs	144 (39.1%)	63 (43.2%)	0.4603
G 3–4 irAEs	17 (4.6%)	10 (6.8%)	0.4222
G1–2 Pneumonitis	38 (10.3%)	42 (28.8%)	< 0.001
G3-4Pneumonitis	6 (1.6%)	6 (4.1%)	0.1755
G1-2Endocrine	41 (11.1%)	11 (7.5%)	0.2888
G3-4Endocrine	0 (0%)	0 (0%)	—
G1-2Dermatitis	33 (9%)	7 (4.8%)	0.1585
G3-4Dermatitis	6 (1.6%)	2 (1.4%)	1
G1-2Hepatitis	45 (12.2%)	14 (9.6%)	0.4883
G3-4Hepatitis	4 (1.1%)	3 (2.1%)	0.4113

## 4 Discussion

Immunotherapy has significantly reshaped the prognosis of NSCLC, with 5-year overall survival rates for stage IV disease now reported to be approximately 20–30% (14–17). However, prognostic assessment in patients with stage IV disease receiving immune checkpoint inhibitor monotherapy or chemo-immunotherapy remains controversial. In the present study, we developed and externally validated an individualized survival prediction model for patients with stage IV NSCLC using pretreatment baseline characteristics from those receiving first-line chemo-immunotherapy. The model incorporated five variables: ECOG performance status, ALP, TP, PLR, and SII. These indicators reflect patient functional status, tumor burden-related biological alterations, nutritional condition, and systemic inflammatory-immune status (18–24), all of which are closely associated with prognosis in advanced NSCLC. By integrating these factors, the model demonstrated improved prognostic discrimination and enabled accurate stratification of patients into low- and high-risk groups based on the median risk score.

TRT is now widely used in the treatment of advanced NSCLC, not only for palliative purposes but also as consolidative or definitive therapy. Beyond symptom control and improved local tumor control, TRT has attracted increasing attention for its immunomodulatory effects in the context of immunotherapy. RT may enhance the efficacy of ICI by increasing tumor antigen release and activating antitumor T-cell responses (25–29). Based on this biological rationale, early clinical investigations have explored the combination of radiotherapy and immunotherapy. Notably, the PEMBRO-RT trial and the MDACC trial reported encouraging signals indicating that the addition of radiotherapy to immune checkpoint blockade may improve clinical outcomes in patients with metastatic NSCLC (9, 10). However, the phase II/III clinical trial NRG-LU002 did not meet its prespecified primary endpoint (30). Radiotherapy in this study primarily consisted of either stereotactic body radiation therapy (SBRT) or hypofractionated radiotherapy (RT) targeting feasible primary or metastatic lesions. However, the trial was terminated early after interim analysis revealed no OS benefit from the addition of radiotherapy. Importantly, the negative findings from NRG-LU002 should not be interpreted merely as evidence opposing the use of local therapy. Instead, they indicate that, in the context of increasingly effective immunotherapy-based systemic treatment, local therapy may be most appropriately incorporated through a selective, biologically informed strategy rather than applied broadly across unselected patient populations.

Notably, several studies have suggested that, among patients with stage IV NSCLC receiving chemo-immunotherapy, the most common site of first progression is the primary lung lesion (31–33). Among patients achieving only stable disease as their best response, the risk of subsequent progression may exceed 75% (33), implying that more aggressive local control may have important clinical value in the immunotherapy era. Accordingly, we developed an OS risk model based on pretreatment clinical variables and further evaluated the effect of TRT across different risk groups to identify patients most likely to benefit and support more individualized treatment strategies in stage IV NSCLC.

Our results indicate that the association between TRT and survival may vary according to model-defined prognostic risk. Among patients classified as low risk, TRT was associated with significantly improved OS, whereas no evident survival advantage was observed among those classified as high risk. The statistically significant interaction between treatment and risk group further supported potential heterogeneity in the association between TRT and OS across prognostic strata. Collectively, these findings imply that the variables included in the model may provide information beyond baseline prognostic estimation by also distinguishing clinically relevant subgroups with differing likelihoods of benefiting from TRT.

These observations are not necessarily inconsistent with the negative findings from NRG-LU002 because of substantial differences in patient selection and the application of local treatment. NRG-LU002 included patients with no more than three extracranial metastatic sites who had achieved at least stable disease after four cycles of first-line systemic therapy. The trial assessed comprehensive LCT, consisting of radiotherapy and/or surgery, rather than TRT alone. By contrast, our study evaluated a broader population with stage IV disease and identified marked variation in treatment outcomes according to a pretreatment prognostic model that incorporated performance status, nutritional status, and systemic inflammatory markers. Patients in the low-risk group may have had greater physiological reserve, more favorable disease biology, and a sufficiently long expected survival for enhanced thoracic control to be associated with a measurable survival benefit. Conversely, systemic disease progression may have remained the predominant determinant of survival among patients in the high-risk group. Differences in radiation target selection, treatment intent, treatment timing, and biological dose may also have contributed to the divergent results.

The exploratory subgroup analyses provided additional support for the risk-specific pattern identified in the primary analysis. After stratification by best treatment response, TRT was associated with improved OS among low-risk patients in both the complete response/partial response (CR/PR) and stable disease/progressive disease (SD/PD) subgroups. By contrast, no significant association with survival was detected in either corresponding subgroup among high-risk patients. These results indicate that the association between TRT and OS may extend beyond low-risk patients who achieve a favorable response to first-line systemic therapy. They also suggest that model-defined risk stratification may offer clinically relevant information beyond that provided by radiographic response alone.

A comparable pattern emerged when patients were stratified according to TRT intent. Within the low-risk group, OS differed significantly among patients who received no TRT, consolidative TRT, or salvage TRT, and both consolidative and salvage TRT were associated with more favorable survival outcomes than no TRT. In contrast, survival did not differ significantly across the corresponding treatment categories in the high-risk group. These observations suggest that, among patients with a favorable prognostic profile, the potential clinical role of TRT may extend beyond the consolidative setting and merits further evaluation as a local treatment strategy after disease progression. Nevertheless, because salvage TRT was administered following disease progression rather than selected as a baseline treatment strategy, the associated findings are not directly comparable with those for treatments determined at baseline and should be interpreted with appropriate caution.

The BED_10_-stratified analysis also revealed differences according to prognostic risk. Among low-risk patients, OS varied significantly across the no-TRT, BED_10_ ≤ 60 Gy, and BED_10_ > 60 Gy groups, with the numerically most favorable survival observed in the BED_10_ > 60 Gy group. In contrast, no comparable difference in survival was detected across these categories among high-risk patients. These observations indicate that more intensive thoracic local treatment may merit further investigation in appropriately selected patients with favorable prognostic profiles. Nevertheless, the overall three-group comparison primarily demonstrates a survival difference between patients who received TRT and those who did not. It does not establish the superiority of BED_10_ > 60 Gy over BED_10_ ≤ 60 Gy or confirm the presence of a definitive dose–response relationship.

The analysis stratified by metastatic burden was likewise broadly consistent with the primary results. Within the low-risk group, the HR point estimates favored TRT in both the oligometastatic and polymetastatic subgroups, although statistical significance was achieved only in the polymetastatic subgroup. By contrast, TRT was not significantly associated with OS in either metastatic-burden subgroup among high-risk patients. Taken together, the generally consistent patterns observed across strata defined by treatment response, TRT intent, BED_10_, and metastatic burden reinforce the potential clinical utility of the prognostic stratification system developed in this study. These findings also provide a rationale for further investigation of selective, risk-adapted strategies for the use of TRT.

Our safety analysis showed that TRT combined with ICI increased the incidence of grade 1–2 pneumonitis, whereas grade 3–4 pneumonitis remained rare and was not significantly increased, consistent with previous reports (10, 34–38). By contrast, the incidences of overall grade 1–2 immune-related adverse events (irAEs), grade 3 irAEs, endocrine toxicity, dermatitis, and hepatitis were comparable between the two groups. These findings suggest that TRT did not substantially broaden the systemic immune-toxicity spectrum associated with ICI but primarily increased an organ-specific toxicity burden centered on irradiated lung tissue.

The online calculator could facilitate a risk-adapted clinical workflow. Before initiating first-line chemo-immunotherapy, pretreatment ECOG performance status, ALP, total protein, PLR, and SII can be entered into the calculator to generate an individualized risk score. In accordance with previous nomogram-based studies highlighting the need for model validation, calibration assessment, and evaluation of clinical utility before implementation (39), prospective multicenter validation of the fixed model and prespecified cutoff is necessary before incorporation into routine clinical practice.

To our knowledge, this is the first study to explore the benefit of TRT in the context of prognostic stratification after first-line chemotherapy-immunotherapy in patients with stage IV NSCLC. These findings raise the possibility that biologically driven risk stratification may provide additional clinical value beyond conventional oligometastatic classification when selecting candidates for TRT. These findings may help identify patients who merit further multidisciplinary evaluation for TRT and may provide a framework for risk-adapted treatment selection. Nevertheless, several limitations should be acknowledged. First, because of the retrospective study design, the analysis remains vulnerable to selection bias, confounding by indication, and residual confounding despite the use of PSM and time-dependent methods. Although the 6-month landmark time-dependent Cox analysis mitigated potential immortal time bias associated with delayed TRT initiation, it could not eliminate bias resulting from nonrandom treatment allocation.

Second, intercenter variation in TRT intent, timing, technique, dose, fractionation, and target-volume delineation may have influenced the observed treatment effects. Although supplementary analyses stratified by treatment response, TRT pattern, BED10, and metastatic burden yielded generally consistent trends within the low-risk group, these results should be regarded as exploratory, particularly because of the limited sample sizes after PSM.

Third, PD-L1 status was unavailable or incompletely recorded for a substantial proportion of patients. The absence of complete PD-L1 data may have affected baseline comparability, treatment selection, and prognostic model performance. In addition, the retrospective design precluded complete adjustment for potential confounding associated with missing PD-L1 information. Prospective studies incorporating standardized PD-L1 assessment are therefore needed to clarify the influence of immune biomarker status on outcomes associated with TRT.

Finally, treatment-related toxicities were assessed retrospectively. Consequently, adverse events, including radiation pneumonitis and immune-related adverse events, may have been underestimated because of incomplete documentation, inconsistent reporting, or reporting bias.

## 5 Conclusions

This multicenter retrospective study suggests that the association between TRT and survival in patients with stage IV NSCLC treated with first-line chemo-immunotherapy may differ according to prognostic risk. TRT was associated with improved overall survival in low-risk patients, whereas no significant association was observed in high-risk patients. These findings support further evaluation of a selective, risk-adapted TRT strategy.

## Data Availability

The raw data supporting the conclusions of this article will be made available by the authors, without undue reservation.

## Supplementary material

Supplementary Figure 1 Kaplan–Meier curves for tertile-based risk stratification in the training, internal validation, and external validation cohorts.

Supplementary Figure 2 **(A)** Covariate balance before and after propensity score matching in the High-risk group. **(B)** Covariate balance before and after propensity score matching in the low-risk group.

Supplementary Figure 3 Overall survival according to the timing of thoracic radiotherapy initiation in low-risk patients.
