# Supplementary material for: Risk-adapted use of thoracic radiotherapy in stage IV non-small cell lung cancer: a multicenter prognostic model study
Source: Front Oncol. 2026 Aug 26;16:1888208. doi: 10.3389/fonc.2026.1888208 (PMC13553371; doi:10.3389/fonc.2026.1888208)
Supplement: Supplementary file 1 [file Table1.docx]

**Supplementary Table 1** Risk-group distribution and prognostic separation using the fixed cutoff of 103.1 across the training and validation cohorts

| **Cohort** | **Total N** | **Low risk, n (%)** | **High risk, n (%)** | **HR, high vs low (95% CI)** | ***P* value** | **Log-rank P value** |
| --- | --- | --- | --- | --- | --- | --- |
| Training cohort | 200 | 168 (84.0%) | 32 (16.0%) | 2.783 (1.672–4.635) | < 0.001 | < 0.0001 |
| Internal validation cohort | 84 | 55 (65.5%) | 29 (34.5%) | 5.031 (2.625–9.642) | < 0.001 | < 0.0001 |
| External validation cohort | 84 | 62 (73.8%) | 22 (26.2%) | 2.788 (1.336–5.820) | 0.0063 | 0.0044 |

**Supplementary Table 2** Tertile-based risk stratification and overall survival across the training and validation cohorts

| Cohort | Low tertile, n (%) | Intermediate tertile, n (%) | High tertile, n (%) | Intermediate vs low tertile, HR (95% CI); *P* value | High vs low tertile, HR (95% CI); *P* value | HR per one-category increase (95% CI) | *P* for trend | Log-rank *P* value |
| --- | --- | --- | --- | --- | --- | --- | --- | --- |
| Training cohort | 67 (33.5%) | 101 (50.5%) | 32 (16.0%) | 2.381 (1.323–4.284); P = 0.0038 | 4.923 (2.512–9.648); P < 0.001 | 2.207 (1.589–3.064) | < 0.001 | < 0.0001 |
| Internal validation cohort | 21 (25.0%) | 34 (40.5%) | 29 (34.5%) | 1.265 (0.485–3.299); *P* = 0.6308 | 5.889 (2.321–14.944); *P* < 0.001 | 2.929 (1.769–4.849) | < 0.001 | < 0.0001 |
| External validation cohort | 26 (31.0%) | 36 (42.9%) | 22 (26.2%) | 2.115 (0.820–5.451); *P* = 0.1212 | 4.496 (1.650–12.256); *P* = 0.0033 | 2.121 (1.297–3.469) | 0.0027 | 0.0065 |

Tertile thresholds were derived exclusively from the training cohort and applied unchanged to the internal and external validation cohorts. Patients were classified as low tertile (risk score ≤ 83.17), intermediate tertile (83.17 < risk score ≤ 103.1), or high tertile (risk score >103.1). Hazard ratios for the intermediate- and high-tertile groups were estimated using the low-tertile group as the reference. The HR per one-category increase was obtained by modeling the ordered risk strata as an ordinal variable. Because the risk score had a discrete distribution with tied values at the thresholds, the three strata were not necessarily equal in size.

**Supplementary Table 3** Baseline characteristics of the low-risk group before and after propensity score matching

| **Variables** | **Before PSM** | | | **After PSM** | | |
| --- | --- | --- | --- | --- | --- | --- |
|  | **ICI**  **(N = 290)** | **ICI+TRT (N = 108)** | ***P***  **value** | **ICI**  **(N = 98)** | **ICI+TRT**  **(N = 98)** | ***P***  **value** |
| **Age** |  |  | **0.547** |  |  | **0.849** |
| < 70 | 234 (80.7%) | 90 (83.3%) |  | 82 (83.7%) | 81 (82.7%) |  |
| ≥ 70 | 56 (19.3%) | 18 (16.7%) |  | 16 (16.3%) | 17 (17.3%) |  |
| **Sex** |  |  | **0.935** |  |  | **0.470** |
| Female | 58 (20%) | 22 (20.4%) |  | 17 (17.3%) | 21 (21.4%) |  |
| Male | 232 (80%) | 86 (79.6%) |  | 81 (82.7%) | 77 (78.6%) |  |
| **N stage** |  |  | **0.777** |  |  | **0.830** |
| N0-1 | 38 (13.1%) | 13 (12%) |  | 13 (13.3%) | 12 (12.2%) |  |
| N2-3 | 252 (86.9%) | 95 (88%) |  | 85 (86.7%) | 86 (87.8%) |  |
| **T stage** |  |  | **0.347** |  |  | **0.062** |
| T1-2 | 127 (43.8%) | 53 (49.1%) |  | 37 (37.8%) | 50 (51%) |  |
| T3-4 | 163 (56.2%) | 55 (50.9%) |  | 61 (62.2%) | 48 (49%) |  |
| **Oligo** |  |  | **< 0.001** |  |  | **0.280** |
| No | 241 (83.1%) | 66 (61.1%) |  | 71 (72.4%) | 64 (65.3%) |  |
| Yes | 49 (16.9%) | 42 (38.9%) |  | 27 (27.6%) | 34 (34.7%) |  |
| **Liver metastasis** |  |  | **0.361** |  |  | **0.306** |
| No | 266 (91.7%) | 102 (94.4%) |  | 95 (96.9%) | 92 (93.9%) |  |
| Yes | 24 (8.3%) | 6 (5.6%) |  | 3 (3.1%) | 6 (6.1%) |  |
| **Pleural effusion** |  |  | **< 0.001** |  |  | **0.470** |
| No | 236 (81.4%) | 103 (95.4%) |  | 95 (96.9%) | 93 (94.9%) |  |
| Yes | 54 (18.6%) | 5 (4.6%) |  | 3 (3.1%) | 5 (5.1%) |  |
| **Brain metastases** |  |  | **0.498** |  |  | **0.699** |
| No | 252 (86.9%) | 91 (84.3%) |  | 81 (82.7%) | 83 (84.7%) |  |
| Yes | 38 (13.1%) | 17 (15.7%) |  | 17 (17.3%) | 15 (15.3%) |  |
| **Bone metastases** |  |  | **0.270** |  |  | **0.421** |
| No | 187 (64.5%) | 76 (70.4%) |  | 74 (75.5%) | 69 (70.4%) |  |
| Yes | 103 (35.5%) | 32 (29.6%) |  | 24 (24.5%) | 29 (29.6%) |  |
| **Adrenal metastasis** |  |  | **0.117** |  |  | **0.733** |
| No | 259 (89.3%) | 102 (94.4%) |  | 94 (95.9%) | 93 (94.9%) |  |
| Yes | 31 (10.7%) | 6 (5.6%) |  | 4 (4.1%) | 5 (5.1%) |  |
| **Pleurainvolement** |  |  | **0.631** |  |  | **0.723** |
| No | 230 (79.3%) | 88 (81.5%) |  | 77 (78.6%) | 79 (80.6%) |  |
| Yes | 60 (20.7%) | 20 (18.5%) |  | 21 (21.4%) | 19 (19.4%) |  |
| **Basic Disease** |  |  | **0.861** |  |  | **0.884** |
| No | 180 (62.1%) | 66 (61.1%) |  | 59 (60.2%) | 60 (61.2%) |  |
| Yes | 110 (37.9%) | 42 (38.9%) |  | 39 (39.8%) | 38 (38.8%) |  |
| **Smoking** |  |  | **0.173** |  |  | **0.561** |
| No | 102 (35.2%) | 46 (42.6%) |  | 38 (38.8%) | 42 (42.9%) |  |
| Yes | 188 (64.8%) | 62 (57.4%) |  | 60 (61.2%) | 56 (57.1%) |  |
| **ECOG** |  |  |  |  |  |  |
| 0-1 | 290 (100%) | 108 (100%) |  | 98 (100%) | 98 (100%) |  |
| 2 | 0 (0%) | 0 (0%) |  | 0 (0%) | 0 (0%) |  |
| **Histology** |  |  | **0.004** |  |  | **0.774** |
| Adenocarcinoma | 175 (60.3%) | 48 (44.4%) |  | 44 (44.9%) | 46 (46.9%) |  |
| Squamous | 115 (39.7%) | 60 (55.6%) |  | 54 (55.1%) | 52 (53.1%) |  |
| **ALP** |  |  | **0.794** |  |  | **0.692** |
| Low | 250 (86.2%) | 92 (85.2%) |  | 82 (83.7%) | 84 (85.7%) |  |
| High | 40 (13.8%) | 16 (14.8%) |  | 16 (16.3%) | 14 (14.3%) |  |
| **TP** |  |  | **0.008** |  |  | **0.871** |
| Low | 235 (81%) | 74 (68.5%) |  | 73 (74.5%) | 72 (73.5%) |  |
| High | 55 (19%) | 34 (31.5%) |  | 25 (25.5%) | 26 (26.5%) |  |
| **NLR** |  |  | **0.724** |  |  | **0.551** |
| Low | 86 (29.7%) | 34 (31.5%) |  | 37 (37.8%) | 33 (33.7%) |  |
| High | 204 (70.3%) | 74 (68.5%) |  | 61 (62.2%) | 65 (66.3%) |  |
| **PLR** |  |  | **0.437** |  |  | **0.539** |
| Low | 67 (23.1%) | 29 (26.9%) |  | 33 (33.7%) | 29 (29.6%) |  |
| High | 223 (76.9%) | 79 (73.1%) |  | 65 (66.3%) | 69 (70.4%) |  |
| **SII** |  |  | **0.939** |  |  | **1.000** |
| Low | 114 (39.3%) | 42 (38.9%) |  | 42 (42.9%) | 42 (42.9%) |  |
| High | 176 (60.7%) | 66 (61.1%) |  | 56 (57.1%) | 56 (57.1%) |  |
| **PNI** |  |  | **0.009** |  |  | **0.881** |
| Low | 142 (49%) | 37 (34.3%) |  | 34 (34.7%) | 35 (35.7%) |  |
| High | 148 (51%) | 71 (65.7%) |  | 64 (65.3%) | 63 (64.3%) |  |
| **AAPR** |  |  | **0.233** |  |  | **0.879** |
| Low | 211 (72.8%) | 72 (66.7%) |  | 66 (67.3%) | 65 (66.3%) |  |
| High | 79 (27.2%) | 36 (33.3%) |  | 32 (32.7%) | 33 (33.7%) |  |
| **PDL-1** |  |  | **0.180** |  |  | **0.271** |
| Negative | 26 (9%) | 16 (14.8%) |  | 7 (7.1%) | 13 (13.3%) |  |
| Positive | 95 (32.8%) | 29 (26.9%) |  | 34 (34.7%) | 27 (27.6%) |  |
| **Unclear** | 169 (58.3%) | 63 (58.3%) |  | 57 (58.2%) | 58 (59.2%) |  |

**Supplementary Table 4** Baseline characteristics of the high-risk group before and after propensity score matching

| **Variables** | **Before PSM** | | | **After PSM** | | |
| --- | --- | --- | --- | --- | --- | --- |
|  | **ICI**  **(N = 78)** | **ICI+TRT**  **(N = 38)** | ***P***  **value** | **ICI**  **(N = 27)** | **ICI+TRT**  **(N = 27)** | ***P***  **value** |
| **Age** |  |  | **0.047** |  |  | **0.715** |
| < 70 | 52 (66.7%) | 32 (84.2%) |  | 23 (85.2%) | 22 (81.5%) |  |
| ≥ 70 | 26 (33.3%) | 6 (15.8%) |  | 4 (14.8%) | 5 (18.5%) |  |
| **Sex** |  |  | **0.951** |  |  | **0.735** |
| Female | 14 (17.9%) | 7 (18.4%) |  | 5 (18.5%) | 6 (22.2%) |  |
| Male | 64 (82.1%) | 31 (81.6%) |  | 22 (81.5%) | 21 (77.8%) |  |
| **N stage** |  |  | **0.678** |  |  | **1.000** |
| N0-1 | 12 (15.4%) | 7 (18.4%) |  | 4 (14.8%) | 4 (14.8%) |  |
| N2-3 | 66 (84.6%) | 31 (81.6%) |  | 23 (85.2%) | 23 (85.2%) |  |
| **T stage** |  |  | **0.411** |  |  | **0.584** |
| T1-2 | 35 (44.9%) | 14 (36.8%) |  | 11 (40.7%) | 13 (48.1%) |  |
| T3-4 | 43 (55.1%) | 24 (63.2%) |  | 16 (59.3%) | 14 (51.9%) |  |
| **Oligo** |  |  | **0.016** |  |  | **0.761** |
| No | 68 (87.2%) | 26 (68.4%) |  | 19 (70.4%) | 20 (74.1%) |  |
| Yes | 10 (12.8%) | 12 (31.6%) |  | 8 (29.6%) | 7 (25.9%) |  |
| **Liver metastasis** |  |  | **0.109** |  |  | **0.313** |
| No | 69 (88.5%) | 37 (97.4%) |  | 27 (100%) | 26 (96.3%) |  |
| Yes | 9 (11.5%) | 1 (2.6%) |  | 0 (0%) | 1 (3.7%) |  |
| **Pleural effusion** |  |  | **0.388** |  |  | **0.340** |
| No | 58 (74.4%) | 31 (81.6%) |  | 19 (70.4%) | 22 (81.5%) |  |
| Yes | 20 (25.6%) | 7 (18.4%) |  | 8 (29.6%) | 5 (18.5%) |  |
| **Brain metastases** |  |  | **0.016** |  |  | **0.552** |
| No | 72 (92.3%) | 29 (76.3%) |  | 25 (92.6%) | 26 (96.3%) |  |
| Yes | 6 (7.7%) | 9 (23.7%) |  | 2 (7.4%) | 1 (3.7%) |  |
| **Bone metastases** |  |  | **0.185** |  |  | **0.785** |
| No | 37 (47.4%) | 23 (60.5%) |  | 13 (48.1%) | 14 (51.9%) |  |
| Yes | 41 (52.6%) | 15 (39.5%) |  | 14 (51.9%) | 13 (48.1%) |  |
| **Adrenal metastasis** |  |  | **0.565** |  |  | **1.000** |
| No | 65 (83.3%) | 30 (78.9%) |  | 21 (77.8%) | 21 (77.8%) |  |
| Yes | 13 (16.7%) | 8 (21.1%) |  | 6 (22.2%) | 6 (22.2%) |  |
| **Pleural involvement** |  |  | **0.543** |  |  | **1.000** |
| No | 62 (79.5%) | 32 (84.2%) |  | 22 (81.5%) | 22 (81.5%) |  |
| Yes | 16 (20.5%) | 6 (15.8%) |  | 5 (18.5%) | 5 (18.5%) |  |
| **Basic Disease** |  |  | **0.800** |  |  | **0.586** |
| No | 43 (55.1%) | 20 (52.6%) |  | 12 (44.4%) | 14 (51.9%) |  |
| Yes | 35 (44.9%) | 18 (47.4%) |  | 15 (55.6%) | 13 (48.1%) |  |
| **Smoking** |  |  | **0.811** |  |  | **1.000** |
| No | 29 (37.2%) | 15 (39.5%) |  | 10 (37%) | 10 (37%) |  |
| Yes | 49 (62.8%) | 23 (60.5%) |  | 17 (63%) | 17 (63%) |  |
| **ECOG** |  |  | **0.272** |  |  | **0.715** |
| 0-1 | 54 (69.2%) | 30 (78.9%) |  | 22 (81.5%) | 23 (85.2%) |  |
| 2 | 24 (30.8%) | 8 (21.1%) |  | 5 (18.5%) | 4 (14.8%) |  |
| **Histology** |  |  | **0.295** |  |  | **0.276** |
| Adenocarcinoma | 45 (57.7%) | 18 (47.4%) |  | 15 (55.6%) | 11 (40.7%) |  |
| Squamous | 33 (42.3%) | 20 (52.6%) |  | 12 (44.4%) | 16 (59.3%) |  |
| **ALP** |  |  | **0.114** |  |  | **0.552** |
| Low | 15 (19.2%) | 3 (7.9%) |  | 2 (7.4%) | 1 (3.7%) |  |
| High | 63 (80.8%) | 35 (92.1%) |  | 25 (92.6%) | 26 (96.3%) |  |
| **TP** |  |  | **0.221** |  |  |  |
| Low | 75 (96.2%) | 38 (100%) |  | 27 (100%) | 27 (100%) |  |
| High | 3 (3.8%) | 0 (0%) |  | 0 (0%) | 0 (0%) |  |
| **NLR** |  |  | **0.624** |  |  | **1.000** |
| Low | 13 (16.7%) | 5 (13.2%) |  | 4 (14.8%) | 4 (14.8%) |  |
| High | 65 (83.3%) | 33 (86.8%) |  | 23 (85.2%) | 23 (85.2%) |  |
| **PLR** |  |  | **0.455** |  |  | **1.000** |
| Low | 2 (2.6%) | 2 (5.3%) |  | 1 (3.7%) | 1 (3.7%) |  |
| High | 76 (97.4%) | 36 (94.7%) |  | 26 (96.3%) | 26 (96.3%) |  |
| **SII** |  |  | **0.750** |  |  | **0.685** |
| Low | 12 (15.4%) | 5 (13.2%) |  | 4 (14.8%) | 3 (11.1%) |  |
| High | 66 (84.6%) | 33 (86.8%) |  | 23 (85.2%) | 24 (88.9%) |  |
| **PNI** |  |  | **0.518** |  |  | **0.783** |
| Low | 50 (64.1%) | 22 (57.9%) |  | 16 (59.3%) | 15 (55.6%) |  |
| High | 28 (35.9%) | 16 (42.1%) |  | 11 (40.7%) | 12 (44.4%) |  |
| **AAPR** |  |  | **0.159** |  |  | **0.444** |
| Low | 66 (84.6%) | 28 (73.7%) |  | 24 (88.9%) | 22 (81.5%) |  |
| High | 12 (15.4%) | 10 (26.3%) |  | 3 (11.1%) | 5 (18.5%) |  |
| **PD-L1** |  |  | **0.254** |  |  | **0.477** |
| Negative | 6 (7.7%) | 6 (15.8%) |  | 2 (7.4%) | 5 (18.5%) |  |
| Positive | 20 (25.6%) | 6 (15.8%) |  | 7 (25.9%) | 6 (22.2%) |  |
| **Unclear** | 52 (66.7%) | 26 (68.4%) |  | 18 (66.7%) | 16 (59.3%) |  |

**Supplementary Table 5** Absolute OS differences and exploratory NNT-like estimates in the propensity score-matched low-risk cohort

| **Time point** | **ICI OS, % (95% CI)** | **ICI+TRT OS, % (95% CI)** | **Absolute OS difference, percentage points** | **Exploratory NNT-like estimate** |
| --- | --- | --- | --- | --- |
| 1 year | 96.9 (90.6–99.0) | 96.9 (90.7–99.0) | 0.0 | Not meaningfully estimable |
| 2 years | 75.1 (63.5–83.5) | 87.6 (78.7–93.0) | 12.5 | 9 |

**Supplementary Table 6** Thoracic radiotherapy characteristics and BED10 distribution according to prognostic risk group

| **Characteristic** | **Low-risk group** | **High-risk group** | ***P* value** |
| --- | --- | --- | --- |
| TRT-treated patients, n | 108 | 38 |  |
| **TRT intent** |  |  | **0.545** |
| Consolidative TRT, n (%) | 83 (76.9%) | 31 (81.6%) |  |
| Salvage TRT, n (%) | 25 (23.1%) | 7 (18.4%) |  |
| BED10, median (IQR), Gy | 72.00 (61.80–75.00) | 70.20 (60.00–72.00) | **0.131** |
| **BED10 category using 60-Gy cutoff** |  |  | **0.430** |
| BED10 ≤60 Gy, n (%) | 27 (25.0%) | 12 (31.6%) |  |
| BED10 >60 Gy, n (%) | 81 (75.0%) | 26 (68.4%) |  |
